# Versatile *Trans*-Replication Systems for Chikungunya Virus Allow Functional Analysis and Tagging of Every Replicase Protein

**DOI:** 10.1371/journal.pone.0151616

**Published:** 2016-03-10

**Authors:** Age Utt, Tania Quirin, Sirle Saul, Kirsi Hellström, Tero Ahola, Andres Merits

**Affiliations:** 1 Institute of Technology, University of Tartu, Tartu, 50411, Estonia; 2 Department of Food and Environmental Sciences, University of Helsinki, Helsinki, Finland; Centro de Biología Molecular Severo Ochoa (CSIC-UAM), SPAIN

## Abstract

Chikungunya virus (CHIKV; genus Alphavirus, family *Togaviridae*) has recently caused several major outbreaks affecting millions of people. There are no licensed vaccines or antivirals, and the knowledge of the molecular biology of CHIKV, crucial for development of efficient antiviral strategies, remains fragmentary. CHIKV has a 12 kb positive-strand RNA genome, which is translated to yield a nonstructural (ns) or replicase polyprotein. CHIKV structural proteins are expressed from a subgenomic RNA synthesized in infected cells. Here we have developed CHIKV *trans*-replication systems, where replicase expression and RNA replication are uncoupled. Bacteriophage T7 RNA polymerase or cellular RNA polymerase II were used for production of mRNAs for CHIKV ns polyprotein and template RNAs, which are recognized by CHIKV replicase and encode for reporter proteins. CHIKV replicase efficiently amplified such RNA templates and synthesized large amounts of subgenomic RNA in several cell lines. This system was used to create tagged versions of ns proteins including nsP1 fused with enhanced green fluorescent protein and nsP4 with an immunological tag. Analysis of these constructs and a matching set of replicon vectors revealed that the replicases containing tagged ns proteins were functional and maintained their subcellular localizations. When cells were co-transfected with constructs expressing template RNA and wild type or tagged versions of CHIKV replicases, formation of characteristic replicase complexes (spherules) was observed. Analysis of mutations associated with noncytotoxic phenotype in CHIKV replicons showed that a low level of RNA replication is not a pre-requisite for reduced cytotoxicity. The CHIKV *trans*-replicase does not suffer from genetic instability and represents an efficient, sensitive and reliable tool for studies of different aspects of CHIKV RNA replication process.

## Introduction

Chikungunya virus (CHIKV) (genus Alphavirus, family *Togaviridae*) is a mosquito-transmitted virus and the causative agent of chikungunya fever, which has affected millions during the 2004–2008 Indian Ocean outbreak, and since 2013 in the Caribbean region and the Americas [1]. There are no approved vaccines or specific antivirals for treatment of CHIKV infection, although several active chemical compounds have been reported and some CHIKV vaccine candidates have entered the early phases of clinical trials [2]. CHIKV has been less studied than the model alphaviruses Semliki Forest virus (SFV) and Sindbis virus (SINV). In several aspects, such as the enzymatic functions or structures of virus-encoded proteins, the similarities between these viruses have been experimentally confirmed [3–6]. However, for interactions with the host, both similarities [7–9] and differences [10,11] have been reported. Hence, knowledge acquired from studies of other Old World alphaviruses does not always apply to CHIKV.

CHIKV has a positive-strand RNA genome of approximately 12 kb in length. The genomic RNA has a 5′ cap0 structure and 3′ poly(A) tail and contains two open reading frames (ORF). In the alphavirus genome, there are four conserved sequence elements (CSEs), three of which are required for RNA replication: the 5′ untranslated region (UTR), a 51-nt replication enhancing element in the beginning of the first ORF, and the end of the 3′ UTR followed by at least 11 adenine residues [12,13]. The fourth CSE represents the core of the subgenomic (SG) promoter, required for synthesis of the mRNA for viral structural proteins [14]. The nonstructural (ns) proteins of alphaviruses are expressed from the genomic RNA as a precursor termed P1234 polyprotein, or as a combination of P123 and P1234 polyproteins [15]. The precursors are absolutely required for proper replicase complex formation [16,17]. The partially processed intermediate P123 and nsP4 form a short-lived early replicase that synthesizes a genome-length negative strand [6,18]. Negative-strand synthesis is linked to the formation of membrane invaginations or spherules, which are the physical sites of alphavirus RNA replication [19]. The complete processing of the ns polyprotein alters the replicase to its late form responsible for synthesizing positive-strand genomic and SG RNAs [16]. For CHIKV, most of the replication complexes (spherules) are located close to the plasma membrane [10], whereas for SFV the spherules are predominantly internalized to the surfaces of modified endo- and lysosomal compartments and form vesicular structures termed type I cytopathic vesicles (CPV) [20].

The alphavirus ns proteins have several known enzymatic functions: nsP1 acts as the viral RNA capping enzyme [21], nsP2 is an RNA triphosphatase, NTPase, helicase and protease [3], and nsP4 the core viral RNA-dependent RNA polymerase [22]. A significant amount of each ns protein is located outside of replicase complexes. Individual nsP1 is mostly attached to the inner surface of the plasma membrane and has been shown to counter-act the antiviral effects of tetherin [23]. Individual nsP2 is located both in the nucleus and in the cytoplasm and, for Old World alphaviruses (CHIKV included), is central for counter-acting host antiviral responses [24–26] as well as for suppression of cellular transcription and translation [27,28]. nsP3 is known to interact with various cellular proteins and poly(ADP)ribose [5,8,29,30]. Even nsP4, which is present in infected cells in very low quantities, has been shown to inhibit host antiviral pathways [11]. The ns proteins have a very low tolerance for modifications of their N-termini: substitutions, deletions or additions of even a single amino acid residue have been shown to have serious effects on the functionality of the proteins [3,31–33]. To date, in the context of infectious virus genomes only nsP2 and nsP3 can be engineered to contain large inserts (such as fluorescent proteins or luciferases) in different positions [25,34–37]. In contrast, nsP4 has only been tagged with small 3xFLAG-tag fused to the C-terminus [38].

During virus replication, the expression of ns proteins is coupled with RNA replication. This generates a negative feedback loop for mutations reducing RNA replication. As a result, such mutations are almost always reverted, pseudoreverted and/or compensated by second-site mutations [31,39–41], excluding the possibility to analyze their direct effect on replicase activity. In order to enable such studies, replicase production must be uncoupled from RNA replication. The alphavirus replicases can replicate and transcribe defective interfering RNAs generated in infected cells [42] or artificially constructed RNAs such as helper RNAs [43]. This has allowed construction of *trans*-replication systems so far described for SINV and SFV. These systems have been used to study the biogenesis of spherules [44,45] and for analysis of biological effects of mutations in the ns proteins [19]. Furthermore, SFV *trans*-replication system has uncovered several new biological properties. First, it was found that the size of alphavirus spherules is determined by the length of replicating RNA [46]. Second, SFV replicase was found to generate abundant interferon-inducing RNAs using cellular RNAs as templates [47]. Similar *trans*-complementation systems have also been successfully used to study the composition of the replicase complexes of flaviviruses, another group of positive-strand RNA viruses [48,49].

Here we describe the construction, properties and use of CHIKV *trans*-replication systems. These systems were successfully used to develop CHIKV replicases and replicons containing enhanced green fluorescent protein (EGFP) insertion in nsP1 or with peptide tags attached to the C-terminus of nsP4. The tagged *trans*-replicases enabled efficient spherule formation confirming that the essential steps associated with RNA replication were correctly reproduced in this system. The effects of mutations, associated with noncytotoxic phenotype of CHIKV, on the *trans*-replicase were similar to those observed in the context of replicons and genomes. Thus, CHIKV *trans*-replication system represents a powerful tool applicable for functional studies of CHIKV nsPs, replicase complexes and virus-host interactions; it should also be useful for detailed analysis of inhibitory properties of compounds affecting CHIKV replication.

## Materials and Methods

### Cells and media

All cells were grown at 37°C in 5% CO_2_ atmosphere; 10% fetal bovine serum, 100 U/mL penicillin and 0.1 mg/mL streptomycin were added to growth media. BHK-21 cells (ATCC CCL-10) were grown in Glasgow's Minimal Essential Medium (GMEM, Gibco) containing 2% tryptose phosphate broth and 200 mM HEPES. BSR T7/5 (hereafter BSR) cells, a derivative of BHK-21 cells stably expressing T7 RNA polymerase [50], were cultured in the same medium supplemented with 1 mg/mL of G418 for selection of T7 RNA polymerase expression. Huh7 cells were grown in Dulbecco’s modified Eagle's medium (DMEM, Gibco). COP-5 murine fibroblasts [51] and U2OS (human bone osteosarcoma, ATCC HTB-96) cells were maintained in Iscove's Modified Dulbecco's Medium (IMDM, Gibco) containing L-glutamine.

### Construction of expression vectors for CHIKV replicase

CHIKV sequences were based on the isolate LR2006 OPY1 representing the East Central South African genotype. The sequence encoding for ns polyprotein was optimized according to human codon usage (hereafter this coding sequence is referred to as P1234) and obtained as synthetic DNA (Genscript, USA; other synthetic DNAs were also obtained from the same company). A construct, where only 120 codons from the 5′ end and 52 codons from the 3′ end of CHIKV ns ORF were to correspond to human codon usage, was made for comparison. It contains native codon usage in 93% of ns polyprotein encoding region and was designated P1234-NAT.

**Fig 1:**
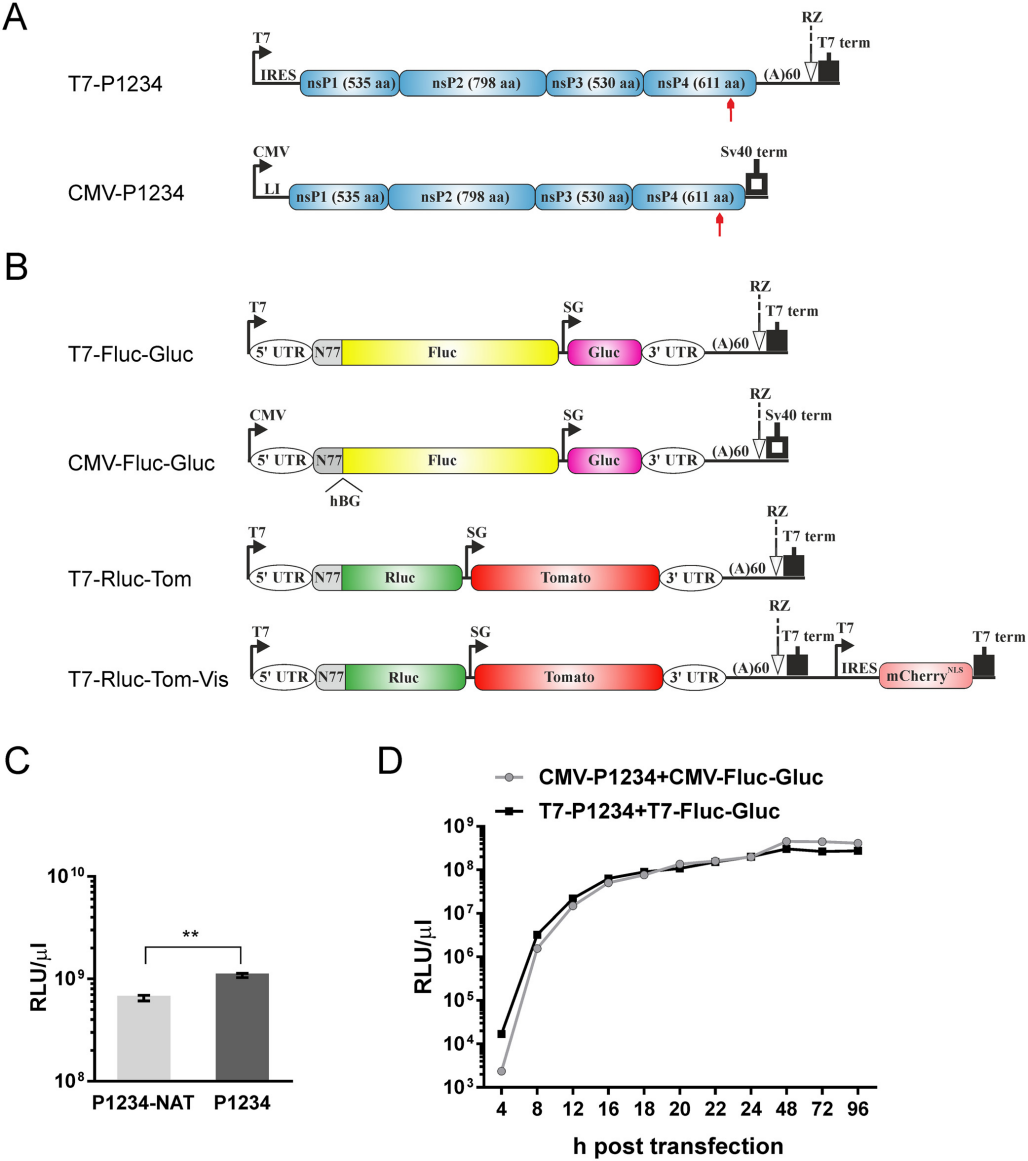
Construction of *trans*-replication systems. **(A)** Schematic presentation of T7- and CMV promoter-based expression constructs of CHIKV replicase. T7—promoter for T7 RNA polymerase; IRES—EMCV IRES; CMV—CMV promoter; LI—HSV TK leader region with an intron. Arrows below the drawings point to the position of the inactivating GDD to GAA mutation in the catalytic site of nsP4. **(B)** Schematic presentation of T7—and CMV promoter-based constructs expressing template RNAs. The 5′ and 3′ UTRs are from CHIKV; N77—region encoding for the 77 N-terminal amino acid residues of nsP1; SG—CHIKV SG promoter. The position of the human beta globin intron (hBG), removed by splicing, is marked. For both A and B, the antisense strand ribozyme of HDV (RZ), T7 transcription terminator and SV40 late polyadenylation region are shown. The drawings are not in scale. **(C)** U2OS cells were co-transfected with CMV-P1234 + CMV-Fluc-Gluc or CMV-P1234-NAT + CMV-Fluc-Gluc. Gluc activity in the medium was measured at 24 h post transfection. An average of three independent experiments is shown, error bars represent standard deviation. ** designates p<0.01 (Student’s paired t-test). **(D)** BSR cells were co-transfected with T7-P1234 + T7-Fluc-Gluc or CMV-P1234 + CMV-Fluc-Gluc. Aliquots of growth media were collected at the indicated time points and used for measurement of Gluc activity. Each point represents an average from three parallel experiments; error bars are too small to be shown.

In order to express ns polyprotein, two different approaches were used. First, expression of mRNA for ns polyprotein was driven by the RNA polymerase of bacteriophage T7. To achieve efficient ns polyprotein translation from non-capped transcripts, the P1234 was placed under control of the internal ribosomal entry site (IRES) of encephalomyocarditis virus (EMCV) [52]. To increase the stability of mRNAs, the IRES-P1234 cassette was flanked by the 5′ and 3′ UTRs from mRNA encoding for human beta actin. 60 adenine residues were added to the 3′ end of the construct; this sequence was followed by the antisense strand ribozyme from hepatitis delta virus (HDV) and terminator for T7 RNA polymerase. The sequence containing all these elements was obtained as synthetic DNA and cloned into pUC57Kan plasmid; the final construct was designated T7-P1234 (Fig 1A). Second, expression of mRNA for ns polyprotein was triggered by the immediate early promoter of human cytomegalovirus (CMV). In this case, the P1234 and P1234-NAT were cloned into pMC-gtGTU2 vector (FIT Biotech Plc, Finland) that contains the CMV promoter, leader sequence from the thymidine kinase of herpes simplex virus (HSV TK) with an inserted synthetic intron and late polyadenylation signal from simian virus 40 (SV40). The constructs were designated CMV-P1234 (Fig 1A) and CMV-P1234-NAT. In addition, the cassette containing the CMV promoter, P1234 and SV40 polyadenylation signal was inserted into pMC.BESPX minicircle production vector [53] to generate MCC-P1234. In order to obtain polymerase-inactive forms of CHIKV replicase the catalytic Gly-Asp-Asp (GDD) motif in nsP4 was changed to Gly-Ala-Ala (GAA) using PCR-based site-directed mutagenesis. The resulting construct was designated P1234^GAA^.

### Construction of plasmids for expression of template RNAs

A matching set of plasmids, designed to express template RNAs for CHIKV replicase from T7 and CMV promoter-based vectors, was constructed as follows. All template RNAs were designed to contain following elements: 1) the full-length 5′ UTR of CHIKV followed by the region encoding for the 77 N-terminal amino acid residues of nsP1 (this region contains the 51-nt CSE); 2) sequence corresponding to the SG promoter and intergenic region of CHIKV (spanning residues -78 to +69 with respect to the start position of SG RNA); 3) the 3′ end (110 residues) of the 3′ UTR of CHIKV followed by 60 adenine residues and HDV antisense strand ribozyme. The elements 1, 2 and 3 were separated from each other by short polylinkers that were subsequently used for insertion of sequences coding for marker proteins.

For T7 promoter-based system the template RNA cassette was placed between the minimal (-17/+1) promoter and terminator of T7 RNA polymerase. Sequence containing all the elements was obtained as synthetic DNA and cloned into pUC57Kan vector. Subsequently, the sequence encoding for *Renilla* luciferase (Rluc) was fused with ORF encoding the 77 N-terminal residues of nsP1 and the sequence encoding for Tomato fluorescent marker protein was inserted under the CHIKV SG promoter; the resulting plasmid was designated T7-Rluc-Tom (Fig 1B). Alternatively, sequences encoding for firefly luciferase (Fluc) and *Gaussia* luciferase (Gluc) were inserted using the same approach, generating T7-Fluc-Gluc (Fig 1B). To obtain plasmid designated as T7-Rluc-Tom-Vis, the second copy of T7 promoter and sequence encoding for mCherry marker protein were included into the T7-Rluc-Tom vector (Fig 1B), as previously described for SFV [19]. For CMV promoter-based system the first residue of template RNA was positioned to the start site of CMV promoter; SV40 late polyadenylation sequence was placed downstream of the cassette for template RNA. The second intron from the human beta globin gene was inserted after residue 303, according to CHIKV LR2006 OPY1 genome numbering. Sequence containing all these elements was obtained as synthetic DNA and cloned into pUC57Kan vector. The markers were inserted as described above; resulting plasmids were designated CMV-Rluc-Tom and CMV-Fluc-Gluc (Fig 1B).

### Insertion of EGFP and peptide tags

Polylinker with sequence 5′-ACTAGTCTCGAGCCCGGG-3′ (sites for SpeI and SmaI underlined) was inserted using PCR-based mutagenesis into the following positions of T7-P1234 and CMV-P1234 plasmids: 1) after codon 492 (site 1A), 497 (site 1B), 516 (site 1C) or 525 (site 1D) of nsP1; 2) after codon 466 (site 2A) or 618 (site 2B) of nsP2; 3) after codon 383 of nsP3. The sequence encoding for EGFP flanked with Gly-Gly linkers was fused with adapters containing sites for SpeI (5′ end) and SmaI (3′ end) endonucleases, amplified by PCR and inserted into the positions listed above. The resulting constructs were designated (either CMV- or T7-) P1^E^234-A, P1^E^234-B, P1^E^234-C, P1^E^234-D, P12^E^34-A, P12^E^34-B, and P123^E^4. Plasmid T7-P123^E^4^GAA^ was obtained from T7-P123^E^4 by changing the Gly-Asp-Asp motif in nsP4 to Gly-Ala-Ala. To fuse EGFP to the C-terminus of nsP4 the flexible linker with sequence Ser-Gly-Gly-Ser-Gly-Gly was added to the N-terminus of EGFP. This fusion was achieved by PCR-based mutagenesis, giving T7-P1234^E^ and CMV-P1234^E^.

To insert sequences recognized by commercially available antibodies and/or purification systems three peptide tags with a similar length were designed: 1) SF-tag consisted of the sequences of Strep- (bold) and 3xFLAG-tag (underlined): **WSHPQFEK**MDYKDHDGDYKDHDIDYKDDDDK; 2) HF-tag consisted of HA- (bold) and 3xFLAG-tag (underlined): **YPYDVPDYA**MDYKDHDGDYKDHDIDYKDDDDK; 3) HS-tag consisted of two copies of HA-tag (bold) followed by Strep-tag (underlined): **YPYDVPDYA**M**YPYDVPDYA**MWSHPQFEK. To attach these tags to the C-terminus of nsP4 a flexible linker with sequence Gly-Gly-Gly-Gly-Ser was added to the N-terminus of the tag. Sequences encoding for such tags were obtained as synthetic DNAs and fused with the 3′ end of the P1234 in T7-P1234 and CMV-P1234 plasmids resulting in (either CMV- or T7-) P1234^SF^, P1234^HF^, and P1234^HS^ expression constructs.

### Introduction of point mutations

Point mutations, resulting in the following changes of encoded nsP2 protein, were made in CMV-P1234 using PCR-based site directed mutagenesis; designations of the constructs are provided in parentheses: Glu117 to Lys (CMV-P12^EK^34), Lys192 to Asn (CMV-P12^KN^34), insertion of Gly-Glu-Glu-Gly-Ser sequence after amino acid residue 647 (CMV-P12^5A^34) and Pro718 to Gly (CMV-P12^PG^34). In addition, two constructs containing nsP2 region harboring combinations of mutations were made: CMV-P12^EKPG^34 and CMV-P12^5APG^34.

### Construction, rescue and analysis of recombinant replicon vectors and viruses

pChikRepl (hereafter referred to as Repl-wt), a plasmid containing the cDNA of CHIKV replicon vector [54], was used for construction of CHIKV replicons expressing nsP1, nsP2 or nsP3 carrying an EGFP insertion. Briefly, PCR-based mutagenesis was used to insert polylinker (5′-ACTAGTCTCGAGCCCGGG-3′) into selected positions of Repl-wt: after codon 516 of nsP1, after codon 486 of nsP2 or after codon 383 of nsP3; sequence encoding for EGFP was inserted as described above. The resulting replicons were designated as Repl-1^E^, Repl-2^E^ and Repl-3^E^.To construct CHIKV replicons expressing nsP4 with SF- or HF-tag at its C-terminus the coding region of nsP4 was fused with that of SF- or HF-tag as described above. In addition, the SG promoter of CHIKV was inactivated by synonymous changes introduced into the region encoding for the 27 C-terminal amino acid residues of nsP4. These replicons were designated Repl-4^SF^ and Repl-4^HF^. pICRES1-SG-Rluc [8] was used to obtain plasmids containing the infectious cDNAs of CHIKV expressing nsP4 with SF- or HF-tag at its C-terminus. The coding regions of SF- or HF-tags were fused to that of nsP4; the native SG promoter was inactivated by synonymous changes as described above. The expression of the mRNA for structural proteins was maintained due to the preservation of copy of the SG promoter (spanning positions -78 to +69 with respect to the SG RNA start site) located upstream of the ORF for structural proteins in the ICRES1-SG-Rluc vector. These recombinant viruses were designated ICRES-4^SF^ and ICRES-4^HF^. All these manipulations were done by using synthetic DNA fragments and subcloning procedures.

Plasmids containing the cDNA of CHIKV replicons or genomes were linearized and transcribed *in vitro* using the mMESSAGE mMACHINE SP6 Transcription kit (Ambion), to obtain RNAs for transfection of BHK-21 cells. These procedures as well as infectious center assay (ICA) were performed as previously described [6].

### Analysis of CHIKV replicase expression and activity in cell culture

The minicircle forms of CMV-P1234 and MCC-P1234 were obtained as described [53]. These minicircles and all plasmid DNAs, used for transfection experiments, were purified using Nucleobond Xtra Midi EF kit (Macherey-Nagel). BHK-21, Huh7, U2OS or BSR cells, grown on 35 mm plates to 90% confluence, were co-transfected with mixtures consisting of 1 μg of plasmid encoding CHIKV replicase and 1 μg of plasmid for expression of corresponding RNA template using Lipofectamine LTX reagent according to the manufacturer's protocols (Invitrogen). COP-5 cells were grown and treated similarly and Lipofectamine 2000 (Invitrogen) was used as transfection reagent. At 18 h post transfection cells were lysed with *Renilla* luciferase assay lysis buffer (Promega) and Fluc and Gluc activities were analyzed using the Dual-Luciferase Reporter Assay kit and Glomax SIS luminometer (Promega); total amount of protein in cell lysates was measured using Bio-Rad protein assay system according to the manufacturer's protocols. In some experiments aliquots of growth media were collected at selected time points and Gluc activity was measured using *Renilla* Luciferase Assay kit (Promega).

### Western blot analysis

Cells were lysed at 24 h (virus/replicon RNA transfected cells) or 18 h (*trans*-replicase transfected cells) post transfection with SDS gel loading buffer (100 mM Tris-HCl pH 6.8, 4% SDS, 20% glycerol, 200 mM dithiothreitol, and 0.2% bromophenol blue), and proteins were separated by 10% SDS-PAGE, transferred to nitrocellulose membranes, and detected using primary antibodies against CHIKV nsP1, nsP2 (recognizes amino acid residues 1–470), nsP3 (recognizes amino acid residues 1–320) and nsP4 (in-house), FLAG (F1804; Sigma-Aldrich), EGFP (in-house) or β-actin (sc-47778; Santa Cruz Biotechnology). The membranes were then incubated with appropriate secondary antibodies conjugated to horseradish peroxidase (LabAs Ltd, Estonia) and proteins were visualized using ECL Immunoblot Detection kit (GE Healthcare).

### Northern blot analysis

BSR or U2OS cells were co-transfected with plasmid DNAs coding for CHIKV replicase and RNA template as described above. At 18 h post transfection, total RNA was extracted using TRIzol^®^ reagent (Life Technologies). Equal amounts of total RNAs (1 μg) were denatured for 10 min at 70°C in 2-fold RNA loading dye (Thermo Scientific), cooled on ice and separated on 1% agarose 6% formaldehyde containing denaturing gel using 1xMOPS buffer. RNA was transferred to a Hybond-N+ filter (GE Healthcare) and fixed using a UV Stratalinker 1800 (Stratagene). A digoxigenin (DIG)-labeled RNA, complementary to the last 110 residues of the 3′ UTR region of CHIKV genome, was generated using a DIG hybridization kit (Roche) and used as a probe. Filters were hybridized overnight; blots were washed and developed according to the manufacturer's protocols (Northern blot with DIG, Roche).

### Confocal microscopy

For immuno-fluorescence imaging, 2x10^4^ U2OS cells were seeded on glass coverslips and incubated overnight at 37°C prior to transfection. For replicon vectors, transfection was performed using 450 ng of *in vitro* transcribed Repl-wt, Repl-1^E^, Repl-2^E^, Repl-3^E^ or Repl-4^SF^ RNAs. For *trans*-replication system, cells were co-transfected using 250 ng of CMV-Rluc-Tom plasmid and 200 ng of CMV-P1234, CMV-P1^E^234, CMV-P12^E^34, CMV-P123^E^4 or CMV-P1234^SF^ plasmids. At 18 h post transfection cells transfected with replicon RNAs or co-transfected with CMV-P1234+CMV-Rluc-Tom or CMV-P1234^SF^+CMV-Rluc-Tom were fixed with 4% paraformaldehyde buffer, quenched with NH_4_Cl and permeabilized with 0.1% Triton X-100. dsRNA and nsP4 with SF-tag were detected using mouse monoclonal antibodies J2 (Scicons, Hungary) or M2 (Sigma-Aldrich) as primary antibodies respectively and Cy5-conjugated anti-mouse antibody (Abcam) as secondary antibody. Samples were mounted with ProLongGold containing DAPI (Molecular Probes). The cells co-transfected with CMV-Rluc-Tom and CMV-P1^E^234, CMV-P12^E^34 or CMV-P123^E^4 were fixed with 4% paraformaldehyde buffer and immediately mounted as described above. In all cases EGFP-tagged ns proteins were detected using EGFP fluorescence. Imaging was done with Leica TCS SP5 confocal microscope (HCX APO 63x/1.30 Corr glycerol objective) or Leica TCS Sp5II HCS A confocal microscope (HCX PL APO 63x/1.2 W Corr/0.17 CS water objective). The images were analyzed using ImageJ software.

### Correlative light and electron microscopy (CLEM)

7.5x10^4^ BSR cells were seeded on 35 mm glass-bottom P35G-2-14-C-Grid dishes (MatTek) and incubated overnight at 37°C. The cells were then co-transfected using 1.25 μg of plasmids expressing the CHIKV replicase and 1 μg of T7-Tom-Rluc (for co-transfection with T7-P123^E^4^GAA^ a T7-Rluc-Tom-Vis was used instead). At 16 h post transfection the cells were fixed with 2% glutaraldehyde (Sigma-Aldrich) in 0.1 M sodium-cacodylate buffer (NaCac, pH7.4) for 20 min at room temperature, washed three times with 0.1 M NaCac buffer and left in the last wash. The cells were immediately imaged using a Leica TCS Sp5II HCS A confocal microscope with the HC PL APO 20x/0.7 CS (air) objective lens. Fluorescent and differential interference contrast (DIC) images of cells containing replicating template RNA (determined by presence of Tomato marker) were obtained for each sample except for cells transfected using T7-P123^E^4^GAA^ plasmid; in this case cells expressing both nsP3-EGFP and mCherry were selected instead. The gridded dishes were marked in selected areas where positive cells were spotted during confocal imaging, and prepared for transmission electron microscopy as previously described [55]. Positive cells, identified during fluorescent and DIC imaging, were located for each sample using Jeol JEM-1400 transmission electron microscope. Gatan Orius SC 1000B bottom mounted CCD-camera was used for imaging. All the CLEM experiments were performed twice.

### Statistical analysis

Statistical analysis was carried out using original datasets and GraphPad Prism software. Student’s paired t-test was used for comparison of two sets of data. To compare means of three or more samples one-way ANOVA Sidak’s multiple comparisons test was used.

## Results

### Design of expression constructs for CHIKV replicase and template RNAs

We set out to develop *trans*-replication systems for CHIKV that could be used in multiple cell types, to study basic biology of CHIKV as well as virus-host interactions. So far most alphavirus *trans*-replication studies have utilized bacteriophage T7 RNA polymerase to express the replicase and its template RNA [19,44–46]. An alternative system based on cellular RNA polymerase II promoter has been described [47] but its properties have not been analyzed in detail. Therefore it was of interest to perform head-to-head comparison of T7- and RNA polymerase II promoter-based CHIKV *trans*-replication systems.

The design of a cassette for T7 promoter-based expression of CHIKV ns polyprotein is shown in Fig 1A. In order to increase its stability, the expressed mRNA includes 5′ and 3′ UTR sequences from human beta actin mRNA. As transcripts made by T7 RNA polymerase lack 5′ cap and 3’ poly(A) structures, the EMCV IRES and a poly(A) element of 60 residues were included. The CMV immediate early promoter was used in vectors relying on use of cellular RNA polymerase II (Fig 1A), and the necessary transcription elements were provided in the vector. For both of these replicases, plasmids for expression of template RNAs were constructed (Fig 1B). The common elements, required for CHIKV replicase-mediated replication/transcription of the template RNAs, were: 1) 307 nucleotides from the 5′-end of CHIKV genome; 2) SG promoter; 3) 110 3′ nucleotides of CHIKV RNA followed by 60 adenine residues. The original transcripts from the minimal T7 promoter contain one extra G-residue at the 5′ end. For the CMV promoter, the 5′ end of template RNA was positioned precisely at the start site. To facilitate the nuclear export of CMV promoter derived RNAs, an intron was inserted into the transcription cassette. The basic template elements 1, 2 and 3 were separated from each other by polylinkers, which were used for insertion of coding sequences of marker proteins (Fig 1B).

### CHIKV replicase expressed from a codon-optimized sequence is highly active *in trans*

The codon usage of alphaviruses represents a compromise between codon usages of their vertebrate host and mosquito vectors. For SFV, both native and codon-optimized replicase expression constructs can be used as parts of *trans*-replication systems [45,47]; however they have not been compared directly. Thus, it is possible that increased replicase expression, achieved by codon optimization, may not be optimal in terms of overall RNA replication. On the other hand, codon optimization enables the removal of sequences that resemble splicing signals, which might generate aberrant mRNAs from CMV promoter-based vectors. Thus, we compared fully codon optimized CMV-P1234 with CMV-P1234-NAT, which is mostly native apart from short terminal regions. These constructs, together with the CMV-Fluc-Gluc, were used to transfect U2OS cells. Both constructs triggered high levels of Gluc expression, but the reporter activity achieved with CMV-P1234 was significantly (~1.7-fold) higher (Fig 1C).

To further maximize *trans*-replicase activity, we first compared CMV-P1234, which contains an intron in the leader sequence, with MCC-P1234 lacking introns. In U2OS cells co-transfected with CMV-P1234 + CMV-Fluc-Gluc or with MCC-P1234 + CMV-Fluc-Gluc the former combination yielded 3.2–3.8-fold higher Gluc activity. Second, the expression cassettes in MCC-P1234 and CMV-P1234 vectors can be converted into minicircles devoid of bacterial DNA; this has been shown to increase expression levels [56,57]. Comparison of standard plasmids and the corresponding minicircles in U2OS, BHK21 and COP-5 cells revealed that replicase produced from MCC-P1234 minicircles was 1.7–2.5-fold more effective activator of Gluc expression. In contrast, for CMV-P1234 the beneficial effect of the minicircle form was minimal (≤1.3 fold). Based on the combined data from these experiments it was decided to use a fully codon-optimized sequence encoding for CHIKV replicase (or its mutant forms) in all vectors. For CMV promoter-based system, CHIKV replicase was expressed from standard CMV-P1234 plasmid.

Next, the expression kinetics of Gluc reporter in BSR cells transfected with CMV- and T7 promoter-based systems were compared. For both, Gluc activity was high already at 8 h post transfection and approached a plateau by 16–18 h post transfection (Fig 1D). Based on this, 16 or 18 h post transfection were selected as endpoints for subsequent experiments.

### Expression of Fluc and Gluc markers in different cell lines transfected with CHIKV *trans*-replicase vectors

**Fig 2:**
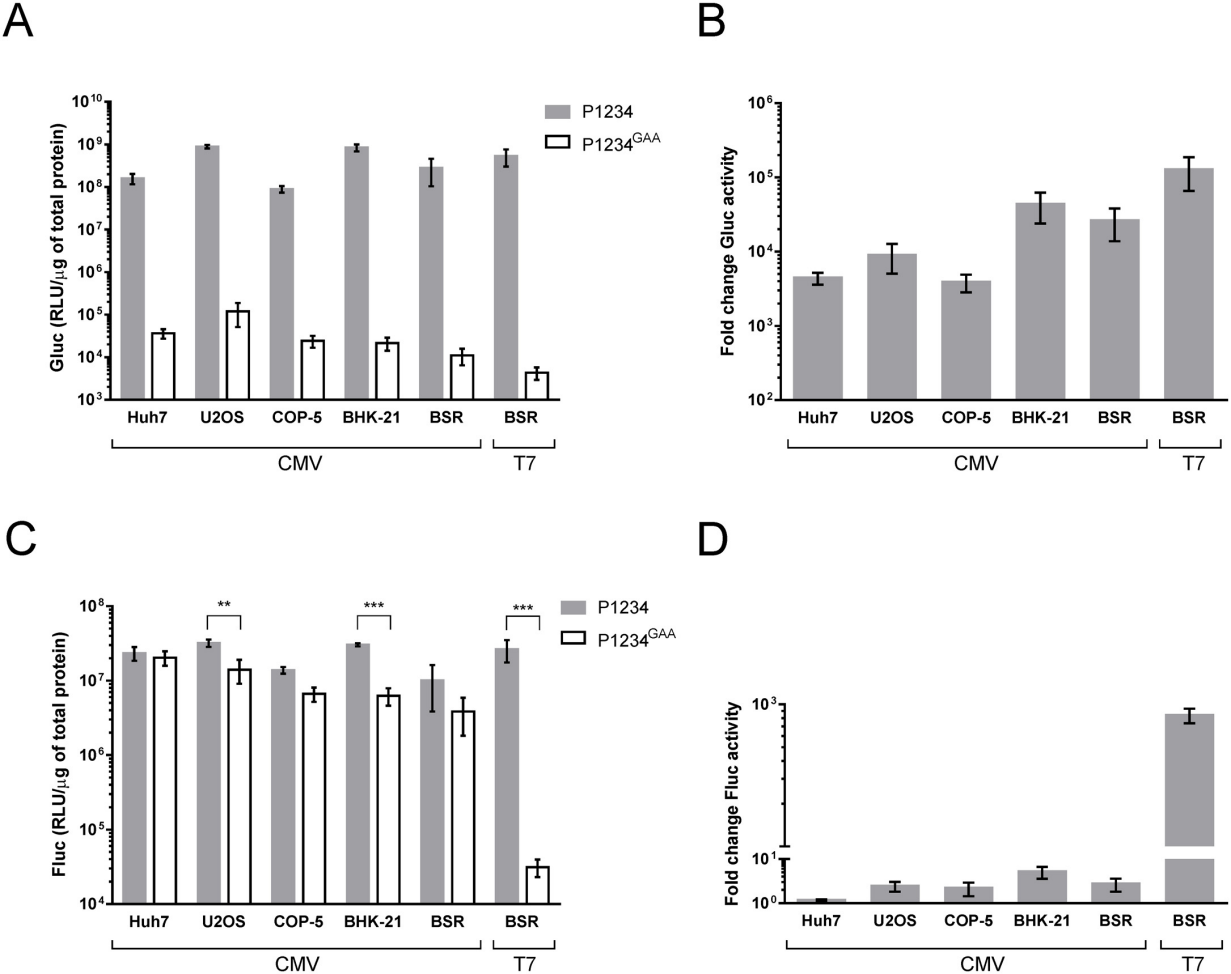
Expression of marker proteins in cell lines transfected with *trans*-replicase vectors. Huh7, U2OS, COP-5, BHK-21 and BSR cells were co-transfected with CMV-P1234 + CMV-Fluc-Gluc (marked as CMV); BSR cells were also co-transfected with T7-P1234 + T7-FLuc-Gluc (marked as T7). Control cells were transfected with T7-FLuc-Gluc or CMV-Fluc-Gluc together with corresponding plasmid encoding for inactive replicase. Cells were lysed at 18 h post transfection. Gluc **(A)** and Fluc **(C)** activities (RLU—relative light unit), normalized to the total protein content in the lysate, are shown. Each column represents an average of three independent experiments; error bars represent standard deviation. All differences between P1234^GAA^ (empty columns) and P1234 (grey columns) were highly significant (p<<0.001); in C ** designates p<0.01 and *** designates p<0.001 (one-way ANOVA Sidak’s multiple comparisons test). **(B)** and **(D)**. The reporter activities generated by the active replicase were normalized to those measured with the inactive controls.

The CMV promoter-based *trans*-replication system was tested in two human (Huh7, U2OS) and three rodent (BHK-21, BSR, COP-5) cell lines; BSR cells were also co-transfected with T7-P1234 + T7-Fluc-Gluc. Gluc is encoded by the second ORF of the template RNA (Fig 1B) and therefore its expression requires the synthesis of SG RNAs. The highest levels of Gluc activity were observed in BHK-21 and U2OS cells but they were very high also in other cell lines (Fig 2A), indicating efficient RNA replication/transcription. The background for Gluc, obtained with an inactive replicase expressed by CMV-P1234^GAA^ or T7-P1234^GAA^, was low and generally a 10^3^−10^5^-fold increase was achieved by *trans*-replication (Fig 2B).

**Fig 3:**
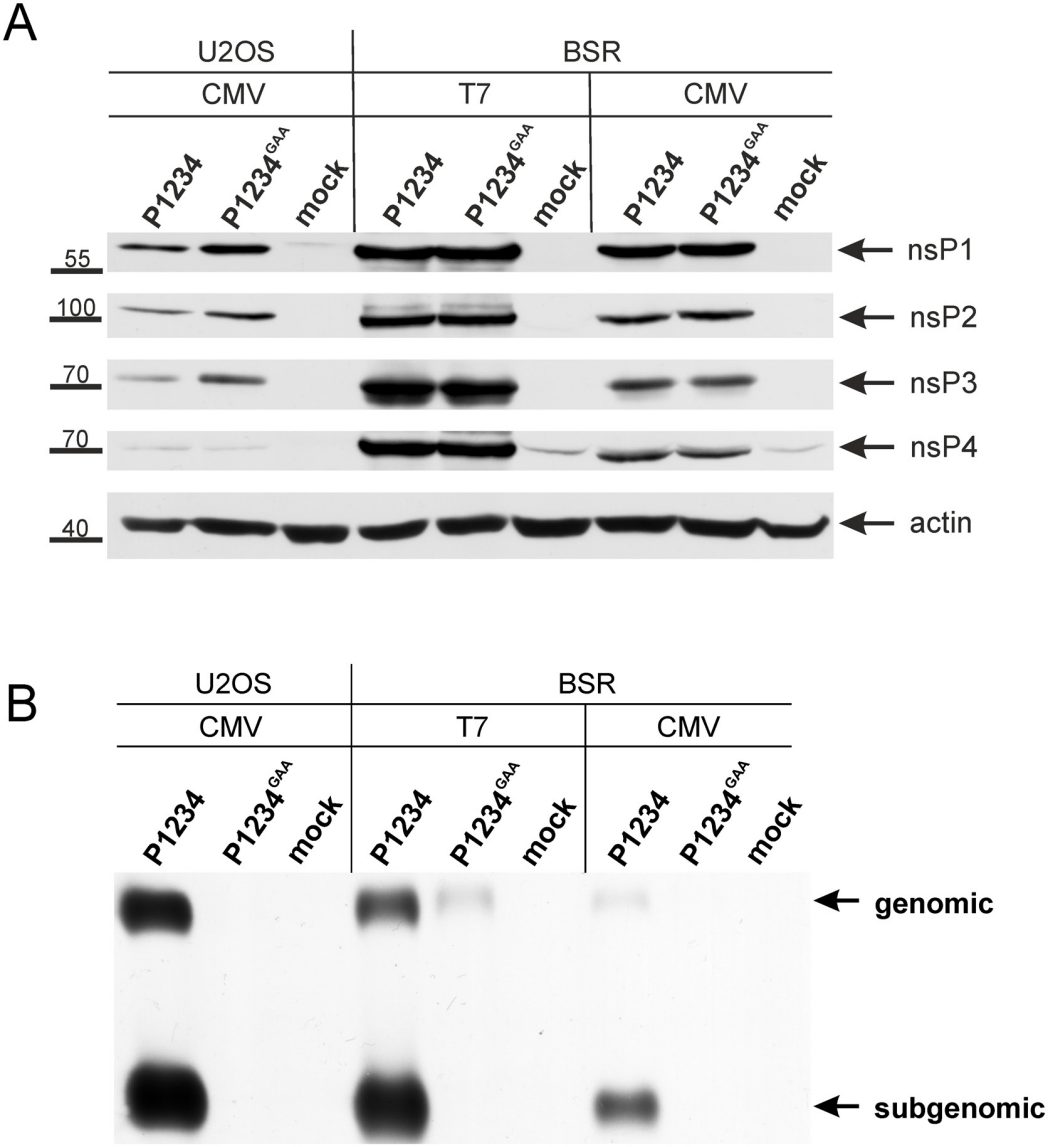
Expression of ns proteins and replication/transcription of template RNA by *trans*-replication systems. U2OS and BSR cells were co-transfected with CMV-P1234 + CMV-Fluc-Gluc or CMV-P1234^GAA^ + CMV-Fluc-Gluc (CMV). In addition, BSR cells were transfected with T7-P1234 + T7-Fluc-Gluc or T7-P1234^GAA^ + T7-Fluc-Gluc (T7); mock-transfected cells were used as controls. Samples were collected at 18 h post transfection. The experiment was repeated three times with similar results; data from one experiment is shown. **(A)** Proteins in cell lysates were analyzed by western blotting using polyclonal antibodies against nsP1, nsP2, nsP3 and nsP4. Antibody against beta actin was used to detect loading control. Sections of gels corresponding to these proteins are shown; appropriate molecular mass markers are indicated on the left. **(B)** RNA was analyzed by northern blotting using a probe complementary to the 3′ UTR of CHIKV. “Genomic” designates the full-length template RNA; note that this RNA is also synthesized by T7 RNA polymerase or cellular RNA polymerase II and is therefore also detectable in the presence of inactive replicases (P1234^GAA^). “Subgenomic” designates RNA synthesized by CHIKV replicase using the SG promoter.

Similarly, high levels of Fluc activities were detected in all the transfected cell lines (Fig 2C). However, as Fluc is encoded by the first ORF of the template RNA, it can also be expressed in the absence of active CHIKV replicase. Coherently, a relatively small increase of Fluc activity was observed for the CMV promoter-based system, when the inactive polymerase was used as a control (Fig 2C and 2D). In contrast, Fluc expression in BSR cells co-transfected with T7-P1234^GAA^ + T7-Fluc-Gluc occurred at a low level, whereas co-transfection of BSR cells with T7-P1234 + T7-Fluc-Gluc resulted in Fluc expression at levels similar to those measured for the CMV promoter-based system (Fig 2C). Since transcription of T7 promoter containing plasmids in BSR cells is efficient [19,46] (Fig 3B), the low levels of Fluc in absence of active CHIKV replicase indicate that such transcripts are poorly translated compared to the transcripts from CMV promoter, likely because they should lack 5′ cap structures. Based on this data U2OS cells were selected as the main cell line for experiments with the CMV promoter-based *trans*-replicase system and inactive polymerase controls were included in all subsequent assays.

### CHIKV *trans*-replication systems amplify the template RNA and generate large amounts of SG RNA

Next, we directly analyzed the presence of CHIKV ns proteins and positive-strand RNAs to compare the T7- and CMV promoter-based systems. CHIKV ns proteins were synthesized both in CMV-P1234 and T7-P1234 transfected cells. The GAA mutation in nsP4 had no negative effect on ns protein expression (Fig 3A). No ns polyproteins were detected indicating that by 18 h post transfection the replicase expression had ceased or that the ns polyproteins were processed too fast to allow their detection by western blotting. The levels of ns proteins depended both on the type of cells and on the design of the *trans*-replication system. In BSR cells the maximal levels of ns protein expression were observed for T7 promoter-based vectors (Fig 3A). This finding could explain why the T7 promoter-based system was generally a more efficient activator of Gluc expression in BSR cells (Fig 2A). However, in some experiments such a difference was not observed (Fig 1D). Furthermore, the levels of CHIKV ns proteins in U2OS cells were clearly lower than in BSR cells (Fig 3A), and yet the expression of Gluc occurred at comparable or even slightly higher level (Fig 2A).

The analysis of viral RNAs revealed that the levels of full-length (“genomic”) transcripts were greatly boosted by active CHIKV replicases (Fig 3B). The original transcripts from CMV-Fluc-Gluc were present at very low levels and undetectable using normal exposure. High Fluc activities in the cells co-transfected with CMV-P1234^GAA^ + CMV-Fluc-Gluc (Fig 2C) indicate that these RNAs were, however, very actively translated. In U2OS cells, the relatively small amount of ns proteins (Fig 3A) and low levels of CMV promoter-derived template RNAs did not seem to limit RNA replication (Fig 3B). In the absence of active replicase the template RNA levels were the highest in BSR cells transfected with T7 promoter-based vectors (Fig 3B). However, also in this case the active replicase caused clear amplification of genomic RNAs and synthesis of large amounts of SG RNA (Fig 3B). Overall, the highest SG RNA levels were seen in U2OS cells (Fig 3B). As the observed SG RNA levels correlated with the Gluc activities in U2OS and BSR cells (Fig 2A) the Gluc activity serves as a good indicator of the efficiency of SG RNA transcription for both CMV and T7 promoter-based *trans*-replication systems. In contrast, Fluc activity can serve as an indicator of template RNA replication only for the T7 promoter-based *trans*-replication system. Therefore hereafter the efficiencies of *trans*-replicases are presented as the normalized Fluc (for template RNA replication) or Gluc (for SG RNA synthesis) activities.

### CHIKV ns proteins, produced by *trans*-replicase vectors, can be tagged using EGFP or epitope tags

Recombinant alphaviruses carrying immunological, affinity, luciferase or fluorescent tags in their ns proteins have been used to study various aspects of the viral life cycle [20,37,38,58,59]. The C-terminal region of nsP3 can accommodate tags in various positions; positions suitable for tag insertion have also been revealed in nsP2 but not in nsP1. SFV or CHIKV with addition of EGFP to the C-terminus of nsP4 are genetically highly unstable and lose inserted EGFP marker (Merits at al., unpublished data). In a *trans*-replication system, however, changes introduced into the replicase proteins cannot be reverted. This should offer possibilities to study the effects of tags and other modifications in ns proteins on the RNA replication as well as on the localization and other functions of these proteins.

**Fig 4:**
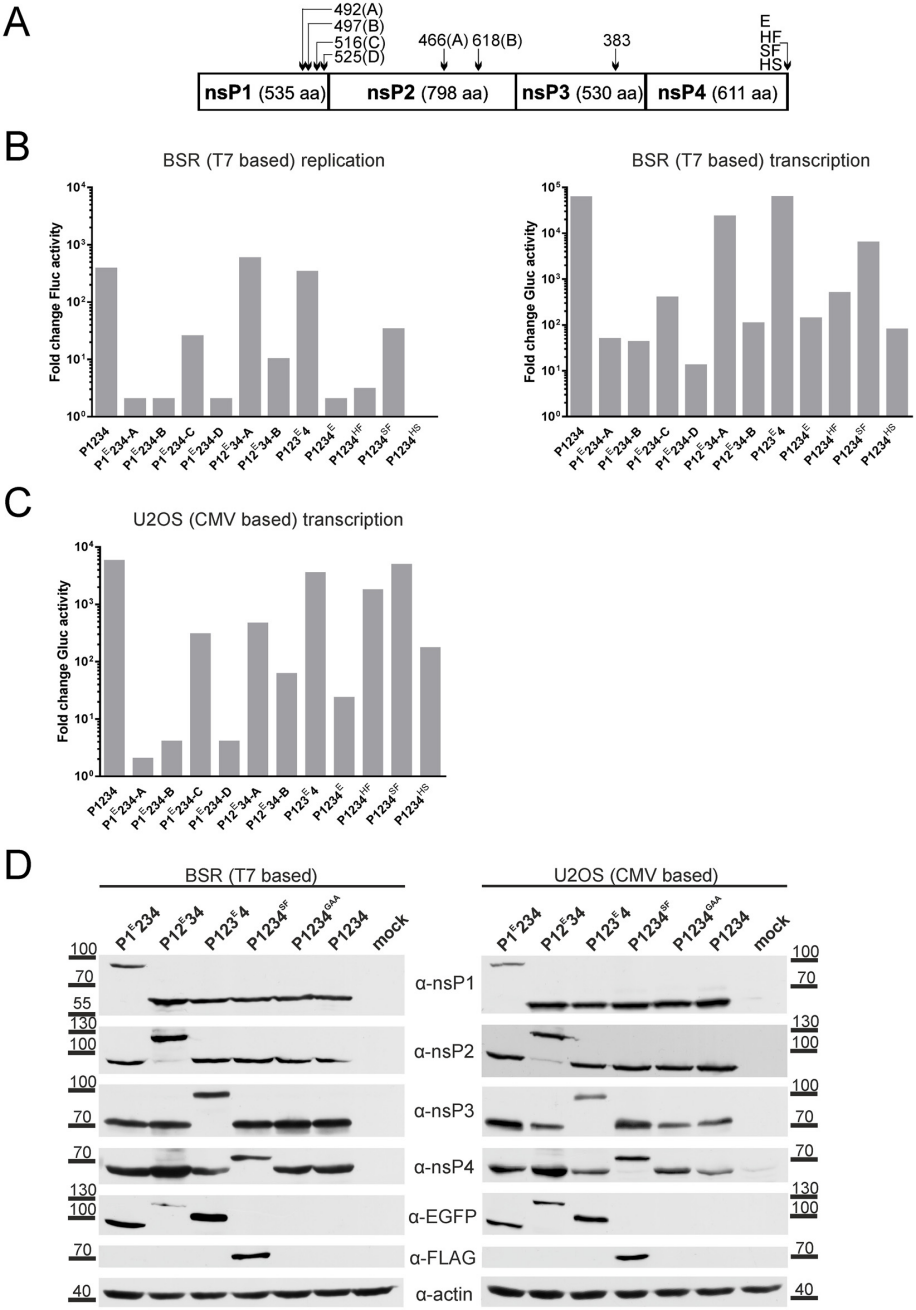
Tagging of replicase expression constructs. **(A)** Positions used for EGFP insertion are indicted by arrows with numbers corresponding to the amino acid residue after which the EGFP was inserted. Tags fused to the C-terminus of nsP4 are designated as follows: E—EGFP; HF—HA-tag+3xFLAG-tag; SF—Strep-tag+3xFLAG-tag; HS—two copies of HA-tag+Strep-tag. **(B)** BSR cells were co-transfected with T7-Fluc-Gluc and T7-P1234 or plasmids expressing tagged versions of ns polyprotein (indicated below the graphs). Cells co-transfected with T7-Fluc-Gluc + T7-P1234^GAA^ served as controls. At 18 h post transfection cells were lysed, and activities of Fluc (left panel) and Gluc (right panel) in the cell lysates were measured. The activities of reporters were normalized to those measured in control cells. Data from one independent reproducible experiment out of three is shown. **(C)** U2OS cells were co-transfected with CMV-Fluc-Gluc and CMV-P1234 or plasmids expressing tagged versions of ns polyprotein. The data is presented as in B. **(D)** BSR (left) and U2OS (right) cells were used to detect expression of ns proteins from T7- and CMV promoter-based vectors, respectively. The constructs used are indicated at the top. Analysis was performed as described for Fig 3A; additionally anti-EGFP and anti-FLAG antibodies were also used for detection of proteins carrying corresponding tags. Data from one independent reproducible experiment out of two is shown.

EGFP was inserted into four different positions located in the C-terminal region of nsP1, into two positions in nsP2 corresponding to those previously used to tag SINV nsP2 [37], into the previously used position in nsP3 [60] and, via short flexible linker, to the C-terminus of nsP4 (Fig 4A). Corresponding T7 promoter-based expression constructs were transfected to BSR cells together with the T7-Fluc-Gluc. Based on Fluc and Gluc activities, it was concluded that the EGFP insertion in nsP3 (P123^E^4) had little to no effect on the template RNA replication/transcription (Fig 4B). The same was observed for EGFP inserted after amino acid residue 466 of nsP2 (P12^E^34-A); in contrast insertion of EGFP after amino acid residue 618 of nsP2 (P12^E^34-B) reduced template RNA replication and transcription at least 100-fold (Fig 4B). The effect of EGFP insertion into nsP1 also strongly depended on the exact position of insertion. Insertions after residues 492 (P1^E^234-A), 497 (P1^E^234-B) or 525 (P1^E^234-D) resulted in prominent reduction of template RNA replication and transcription (Fig 4B). In contrast, insertion after residue 516 (P1^E^234-C) caused milder effects, revealing this to be the most suitable site for EGFP insertion. EGFP fused to the C-terminus of nsP4 allowed for RNA replication and transcription, confirming that nsP4 with such tag was functional. However, the activity of nsP4-EGFP fusion protein was severely compromised: replicase harboring such mutation yielded no more than 1% template RNA replication or transcription compared to wild type replicase (Fig 4B). All these findings were confirmed with CMV promoter-based *trans*-replication systems in U2OS cells (Fig 4C), showing that the effects were cell-type independent. For nsP1 and nsP2, we selected the most active tagged *trans*-replicases (hereafter referred to as P1^E^234 and P12^E^34) for further characterization.

The available antibodies against nsP4 have low efficiency and specificity (note an unspecific band for mock-transfected cells in Fig 3A); coupled with the low abundance it makes compromises detection of nsP4. Additional attempts for tagging of nsP4 with fluorescent proteins were therefore made; unfortunately these too failed to produce satisfactory results. However, it was observed that nsP4 does tolerate short (up to 40 amino acid residues) extensions at its C-terminus. Therefore SF-, HF- and HS-tags of similar (28–32 amino acid residues) length were designed, fused to the C-terminus of nsP4 (Fig 4A) and tested in the context of *trans*-replication systems. Transfection of BSR cells with the T7 promoter-based vectors revealed that the negative effect of the SF-tag on template RNA replication/transcription was the smallest. The HF-tag greatly reduced the activity of the CHIKV *trans*-replication system, and the HS-tag reduced replication of template RNA to a background level (Fig 4B). In U2OS cells transfected with the CMV promoter-based system these effects followed the same pattern, though negative effects of HF- and HS-tags were somewhat less prominent (Fig 4C).

Finally, we wanted to confirm that the tagged *trans*-replicase vectors indeed expressed the correct fusion proteins. For this BSR cells were transfected with T7-P1^E^234, T7-P12^E^34, T7-P123^E^4, T7-1234^SF^, T7-P1234^GAA^ or T7-P1234 vectors; the corresponding set of CMV promoter-based vectors was used to transfect U2OS cells. Western blot analysis revealed an identical set of ns proteins expressed in both cell types. The presence of EGFP in nsP1, nsP2 or nsP3 was evident from the increased sizes of the tagged proteins and by their detection with an anti-EGFP antibody. Similarly, fusion of the SF-tag to nsP4 was detected both by the slightly increased size of the nsP4 and by its recognition with an anti-FLAG antibody (Fig 4D).

### Insertions tested using the *trans*-replication system can be transferred to replicon vectors and infectious genomes

**Fig 5:**
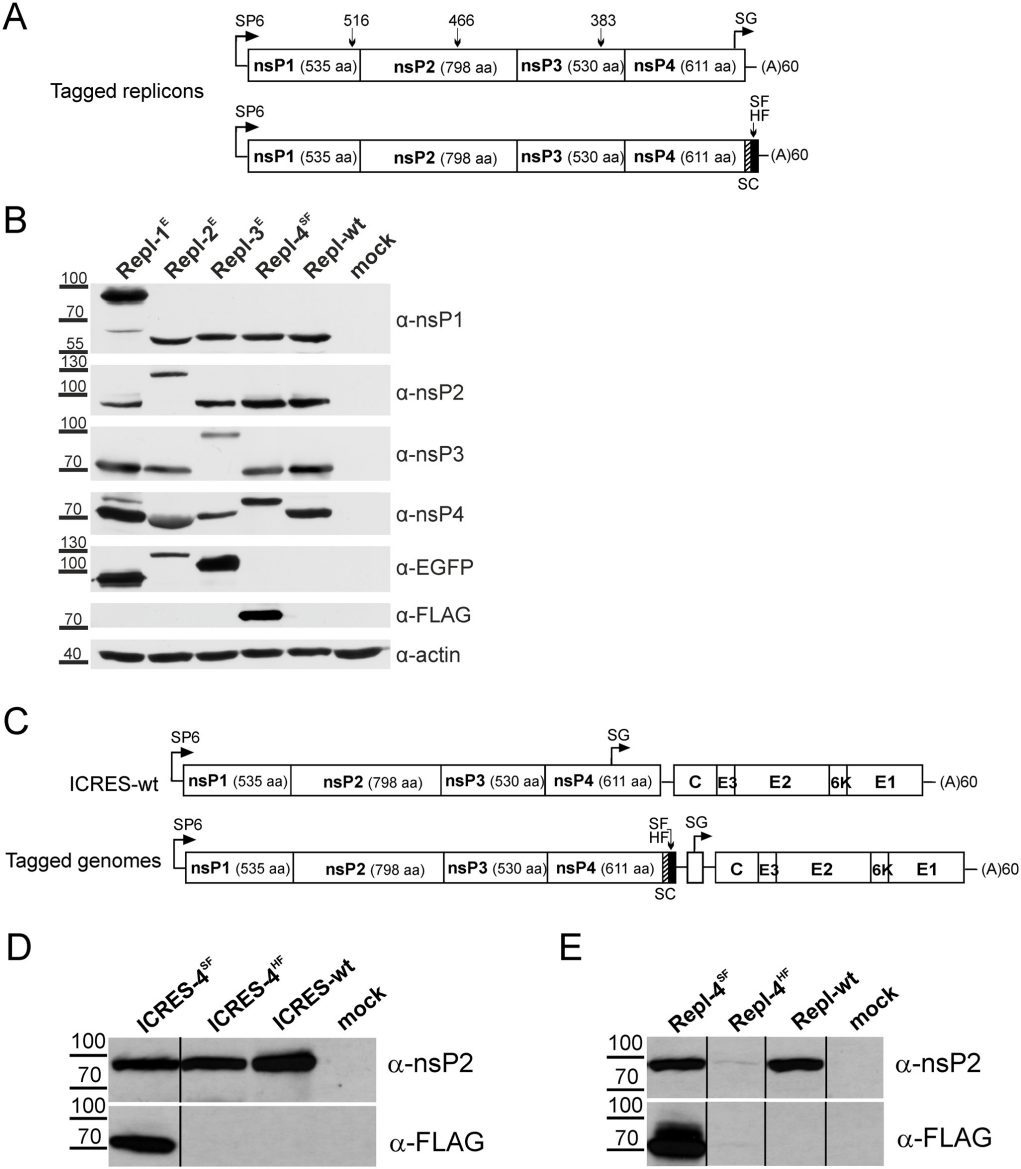
Tagging of ns proteins in the context of CHIKV replicons and genomes. **(A)** Drawing above: sites used for insertion of EGFP into nsP1, nsP2 and nsP3 of CHIKV replicon are shown as in Fig 4A. SG—SG promoter (arrow designates the transcription start site), SP6 –bacteriophage SP6 RNA polymerase promoter. Drawing below: CHIKV replicon encoding for nsP4 with SF- or HF-tag (black) attached to its C-terminus. In these constructs the SG promoter was inactivated by synonymous changes (SC). **(B)** BHK-21 cells were transfected with the indicated *in vitro* synthesized replicon RNAs. Cells were lysed upon detection of prominent cytopathic effects, and the proteins expressed were analyzed as described for Fig 4D. Data from one independent reproducible experiment out of two is shown.**(C)** Schematic representation of genomes of wild type CHIKV (ICRES-wt) and recombinant viruses encoding for nsP4 with tags at the C-terminus of the protein. Designations are the same as on panel A. **(D)** BHK-21 cells were transfected with *in vitro* synthesized ICRES-4^SF^, ICRES-4^HF^ and ICRES-wt RNAs; mock-transfected cells were used as control. Transfected cells were lysed upon detection of prominent cytopathic effects. nsP2 was detected with a corresponding polyclonal antibody and tagged nsP4 was detected with a monoclonal antibody against the FLAG-tag. Data from one independent reproducible experiment out of two is shown.**(E)** BHK-21 cells were transfected with *in vitro* synthesized Repl-4^SF^, Repl-4^HF^ and Repl-wt RNAs and analyzed as described above. Data from one independent reproducible experiment out of two is shown.

**Fig 6:**
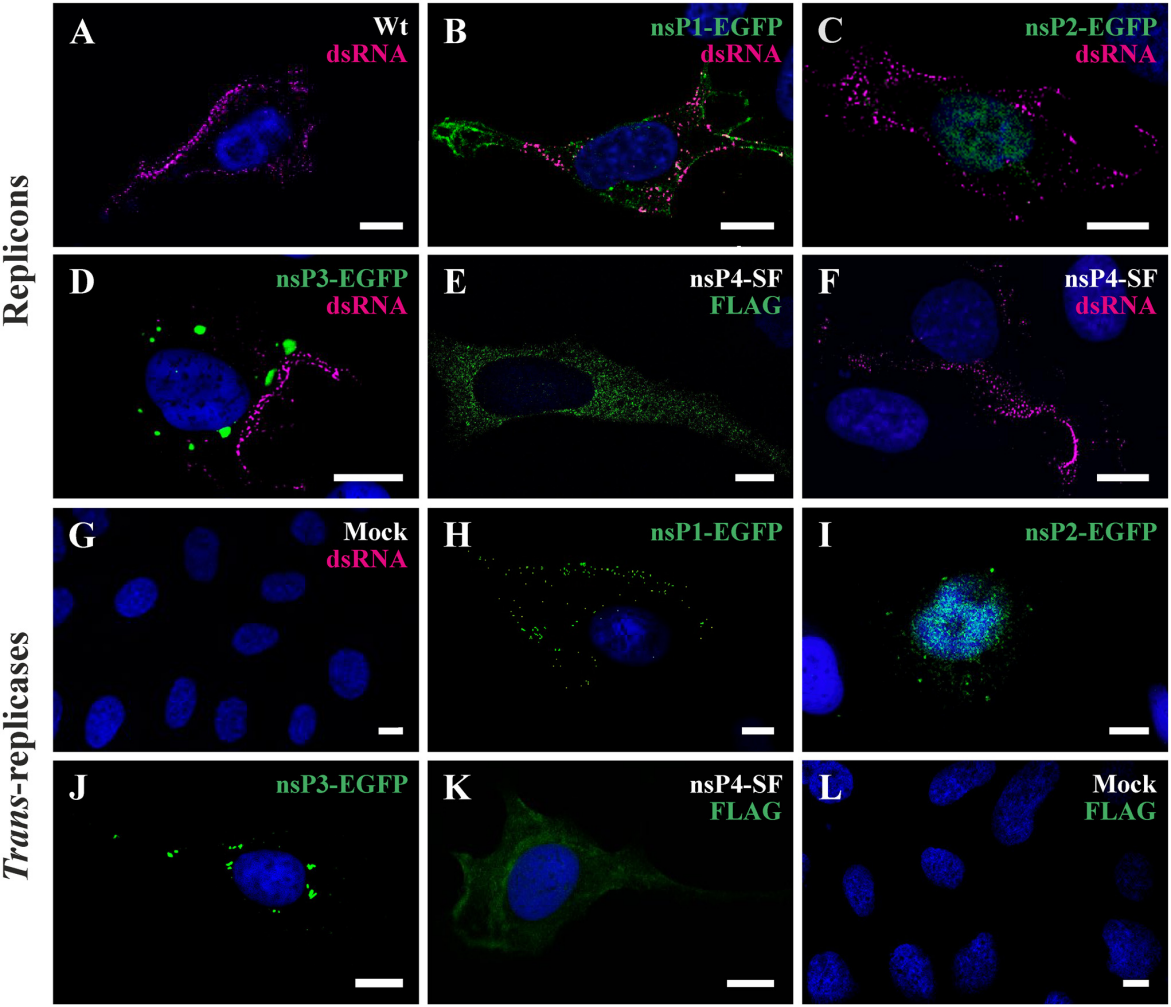
Localization of tagged ns proteins and dsRNA in cells transfected with replicon RNAs or with the corresponding *trans*-replicase constructs. U2OS cells were transfected with *in vitro* synthesized replicon RNAs (A-F), co-transfected with CMV promoter-based replicase and CMV-Rluc-Tom plasmids (H-K) or mock-transfected (G, L). The localization of nsPs (shown in green; with exceptions of panels E and K detected using EGFP fluorescence) and dsRNAs (panels A-D, F; shown in magenta) was analyzed at 18 h post transfection using immunofluorescence microscopy; nuclei were counter-stained with DAPI. Cells characteristic for each construct are shown on the panels. **(A)** Repl-wt, **(B)** Repl-1^E^, **(C)** Repl-2^E^, **(D)** Repl-3^E^ transfected cells; Repl-4^SF^ transfected cells stained with monoclonal antibodies against FLAG-tag **(E)** or dsRNA **(F)**. **(G)** Mock control. **(H)** CMV-P1^E^234 + CMV-Rluc-Tom, **(I)** CMV-P12^E^34 + CMV-Rluc-Tom, **(J)** CMV-P123^E^4 + CMV-Rluc-Tom, **(K)** CMV-P1234^SF^ + CMV-Rluc-Tom transfected cell. **(L)** Mock control. Cells shown on panels H-K were positive for Tomato fluorescence.

First we constructed CHIKV replicon vectors, where the markers were inserted in the same way as in *trans*-replicases (Fig 5A). Transfection of BHK-21 cells with *in vitro* transcribed RNAs of Repl-1^E^, Repl-2^E^, Repl-3^E^, Repl-4^SF^ and Repl-wt resulted in prominent cytotoxic effects indicating efficient RNA replication. In contrast, no such effect was observed for cells transfected with RNAs corresponding to Repl-4^HF^. Cells transfected with RNAs of Repl-1^E^, Repl-2^E^ and Repl-3^E^ also displayed green fluorescence (Fig 6). The analysis of proteins, expressed from Repl-1^E^, Repl-2^E^, Repl-3^E^, Repl-4^SF^ and Repl-wt revealed a picture very similar to that observed for the corresponding *trans*-replicase constructs (compare Figs 5B and 4D). Thus, all these replicons expressed the expected set of ns proteins. Taken together, these experiments revealed that designs developed and evaluated with *trans*-replication systems could be applied to replicating RNAs.

Next, the effects of C-terminal tags in nsP4 were analyzed in the context of full-length CHIKV genome. It was observed that the rescue efficiency of ICRES-4^SF^ (~4.6x10^4^ plaque forming units (PFU)/μg RNA in BHK-21 cells) was only one-log lower than that of ICRES-wt (~5.5x10^5^ PFU/μg RNA). In contrast, the rescue of ICRES-4^HF^ occurred at a much lower level (~9.1x10^2^ PFU/μg RNA). However, the progeny of ICRES-4^HF^ reached a titer of 2.0x10^8^ PFU/mL; even higher than other viruses used in this experiment (1.2x10^8^ PFU/mL for ICRES-wt and 6.3x10^7^ PFU/mL for ICRES-4^SF^). Western blot analysis of infected cells revealed that all three viruses expressed ns proteins roughly at the same level (as an example, nsP2 is shown in Fig 5D). However, only ICRES-4^SF^ but not ICRES-4^HF^ expressed the tagged version of nsP4 (Fig 5D) confirming that the latter virus was genetically unstable and that an overwhelming majority of its progeny had lost the inserted tag (Fig 5E). When the same experiment was performed using replicon vectors it was observed that only Repl-wt and Repl-4^SF^ replicated at a high level and produced large amounts of ns proteins. As expected, Repl-4^SF^ also expressed the tagged form of nsP4. In contrast, expression of nsP2 and tagged nsP4 in Repl-4^HF^ transfected cells occurred at a very low level (Fig 5E). This data unequivocally demonstrates that HF-tag at the C-terminus of nsP4 seriously inhibits virus genome replication.

Interestingly, an additional protein recognized by anti-nsP1 antibodies, most likely originating from replicon RNAs that had lost the inserted marker protein encoding sequence, was also revealed for Repl-1^E^ transfected cells; additional bands, most likely also originating from the instability of Repl-1^E^ replicon, were also detected with nsP2 and nsP4 antibodies (Fig 5B). It could be noted that the replication of template RNA in the cells transfected with T7-P1^E^234-C was less efficient than in cells transfected with T7-P12^E^34, T7-P123^E^4 or T7-P1234^SF^ but more efficient than in cells transfected with T7-P1234^HF^ (Fig 4B, left panel). Thus, there is a clear correlation between the activities of mutant forms of *trans*-replicase and the stability of RNA genomes harboring the same mutations. Furthermore, these findings also highlight one of the main benefits of the *trans*-replication system: the achievement of the genetic stability of mutations in the replicase.

### Visualization of CHIKV ns proteins expressed from tagged replicons and *trans*-replicase vectors

The localization of tagged ns proteins was analyzed using U2OS cells. First, cells were transfected with *in vitro* transcribed replicon RNAs and analyzed at 18 h post transfection (Fig 6A–6F); mock-transfected cells were used as controls (Fig 6G). Staining with antibody against dsRNA revealed that RNA replication occurs predominantly at the plasma membrane both in the Repl-wt (Fig 6A) and tagged replicons (Fig 6B–6D). EGFP-tagged nsP1 was detected at the plasma membrane and showed co-localization with dsRNA (Fig 6B). EGFP-tagged nsP2 was mostly detected in the nucleus (Fig 6C) whereas in Repl-3^E^ transfected cells nsP3-EGFP was found to form large clusters in the cytoplasm (Fig 6D). In cells transfected with Repl-4^SF^, nsP4 visualized with anti-FLAG antibodies was mostly localized diffusely in the cytoplasm (Fig 6E); the RNA replication, however, occurred, as in other replicon RNA transfected cells, at the plasma membrane (Fig 6F).

Next, cells were transfected with the *trans*-replication system (Fig 6H–6K) or mock-transfected (Fig 6L). The *trans*-replication system consists of two plasmids that were not always co-delivered into the same cell. Therefore, the CMV-Rluc-Tomato template, expressing Tomato reporter protein *via* replicase-generated SG RNA, was used to identify cells positive for RNA replication that were subsequently used for imaging. It was found that tagged *trans*-replicase produced fusion proteins localized in the same cellular compartments as their counterparts produced by replicon vectors: nsP1-EGFP localized near the plasma membrane (Fig 6H), nsP2-EGFP was observed mostly in the nucleus (Fig 6I), nsP3-EGFP formed aggregates in the cytoplasm (Fig 6J) and nsP4 expressed from CMV-P1234^SF^ was rather evenly distributed in the cytoplasm (Fig 6K). Similar localization of dsRNA and tagged ns proteins was observed in other cell lines transfected using plasmids of CMV promoter-based *trans*-replicase and in BSR cells transfected using the T7 promoter-based system. Taken together, the localization of tagged ns proteins expressed from CHIKV replicons or by *trans*-replication system was similar to that previously reported for their non-tagged counterparts [8].

### CHIKV *trans*-replication system generates spherules

**Fig 7:**
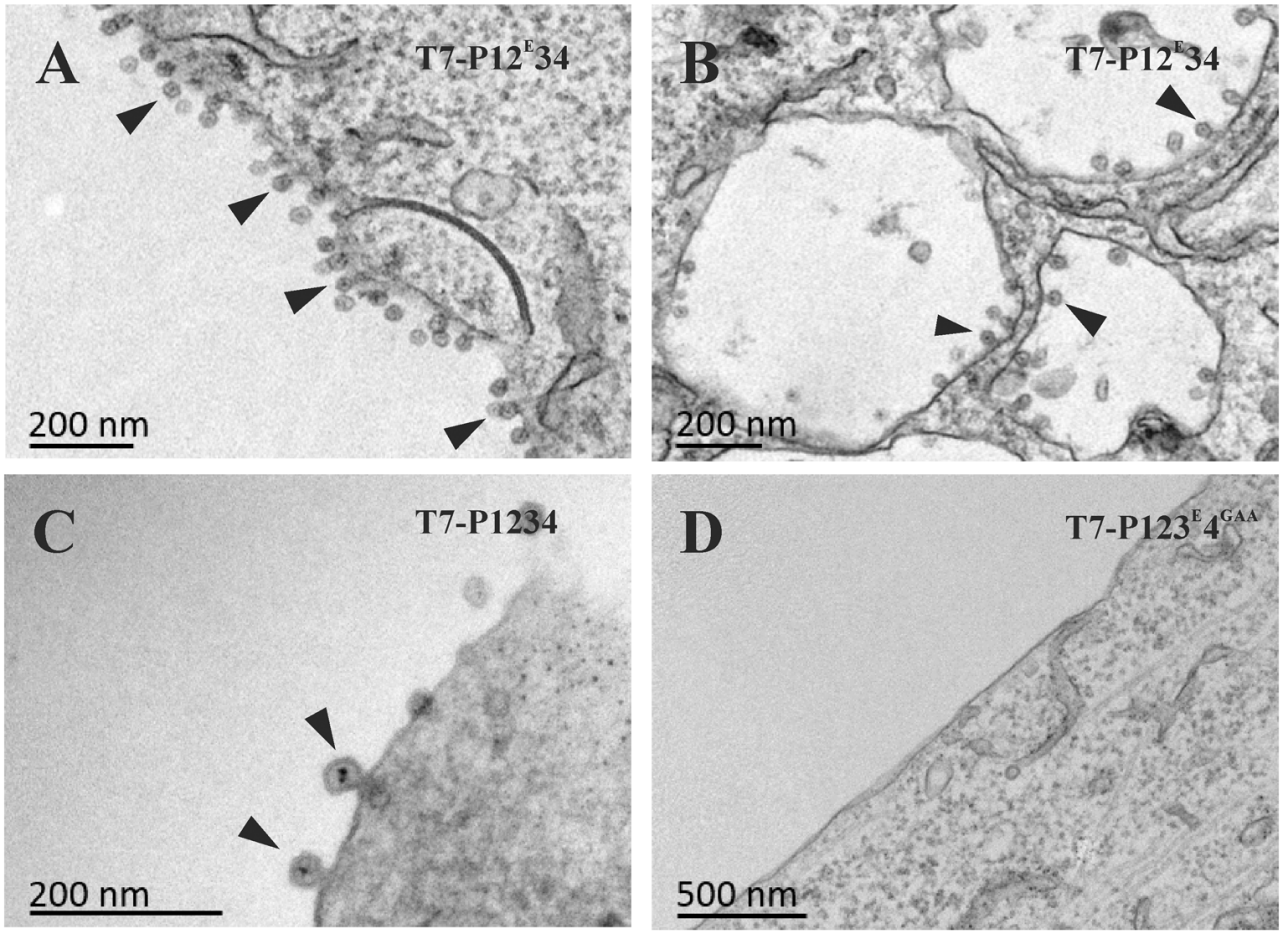
Visualization of spherules in the cells transfected with *trans*-replicase vectors. BSR cells were co-transfected with T7-Rluc-Tom + T7-P12^E^34 **(A, B)**, T7-Rluc-Tom + T7-P1234 **(C)** or T7-Rluc-Tom-Vis + T7-P123^E^4^GAA^ **(D)** plasmids. Cells were fixed and analyzed using CLEM at 16 h post transfection. Spherules (indicated by arrows) were detected in all cells where replication of template RNA was initiated (identified by Tomato fluorescence). Characteristic EM images are shown in panels A-C. No spherules were detected in cells co-transfected with T7-Rluc-Tom-Vis+T7-P123^E^4^GAA^.

To analyze spherule formation using CLEM, BSR cells were co-transfected with T7-Rluc-Tom and T7-P1234, T7-P1^E^234, T7-P12^E^34, T7-P123^E^4 or T7-P1234^SF^. Cells positive for RNA replication were identified as described above and further processed for examination using a transmission electron microscope. Spherules were seen abundantly in all cells where RNA replication occurred and had similar appearance to the spherules formed by SFV *trans*-replicase [45]; no detectable changes were caused by the presence of the tags. For example, in cells transfected with T7-P12^E^34, spherules were observed on the plasma membrane (Fig 7A) and more rarely in CPVs (Fig 7B). The spherules had a characteristic appearance and a ‘neck’ structure that projected towards the cytoplasm; the electron-dense region inside the spherules (Fig 7C) most likely corresponds to the RNA replication intermediate.

To analyze the requirement of RNA replication for spherule formation, BSR cells were also co-transfected with T7-P123^E^4^GAA^ + T7-Rluc-Tom-Vis. The presence of ns proteins produced from T7-P123^E^4^GAA^ was visualized by nsP3-EGFP fluorescence. The presence of T7-Rluc-Tom-Vis in the same cells was confirmed by fluorescence of mCherry that is expressed independent of RNA replication (Fig 1B). Consistent with previous observations made for SFV *trans*-replicase [45] no (Fig 7D) or only few spherule-like structures were observed in these cells. The scarcity of these spherule-like structures led to the conclusion that the formation of true spherules is dependent on RNA replication.

### Analyzing the effects of mutations associated with noncytotoxic phenotype

**Fig 8:**
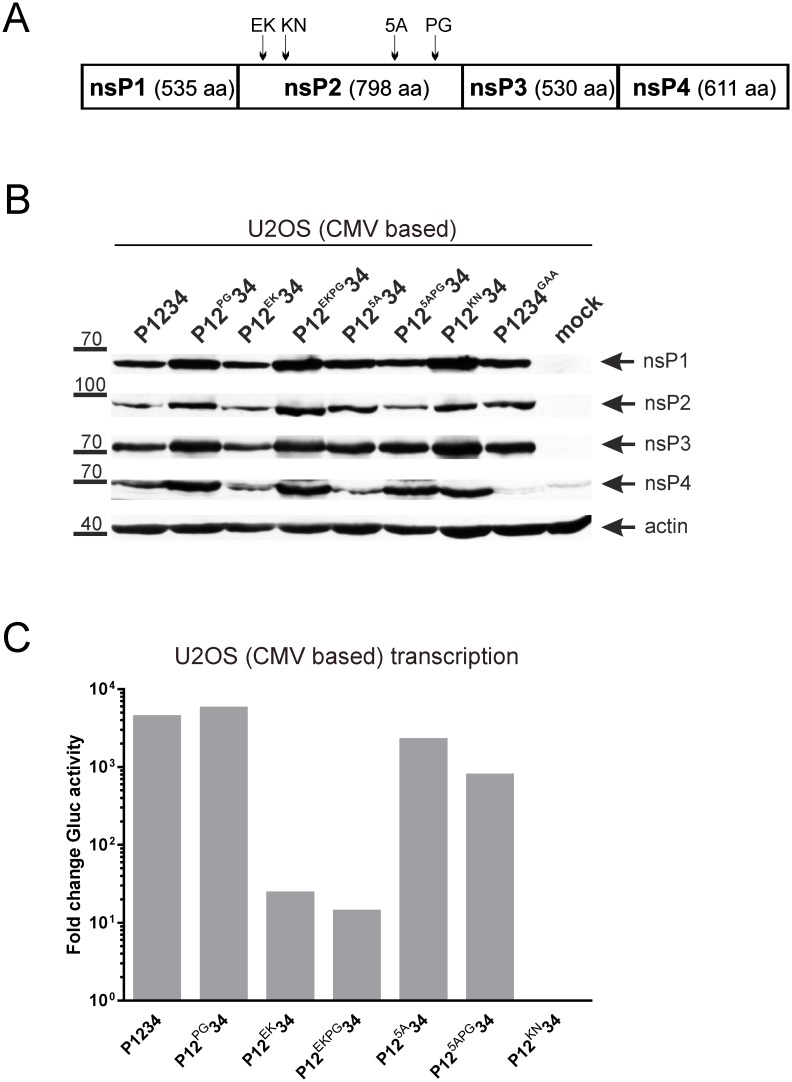
Functional analysis of mutations in nsP2 using CHIKV *trans*-replication system. U2OS cells were co-transfected with CMV-Fluc-Gluc and CMV-P1234 or plasmids expressing mutant versions of ns polyprotein. Cells co-transfected with CMV-Fluc-Gluc + CMV-P1234^GAA^ served as controls. Cells and media were harvested at 18 h post transfection.**(A)** Location of mutations in the coding region of CMV-P1234. **(B)** Expression of ns proteins from CMV-P1234 and its mutant variants (indicated above the graph) was analyzed as described for Fig 3A. **(C)** Activities of Gluc in the growth media were measured and normalized to those measured for control cells. Data from one out of four reproducible independent experiments is shown.

Mutations in the C-terminal region of nsP2 have been shown to reduce the cytotoxicity of alphavirus replicon vectors [25,61,62]. A prominent mutation involved in this phenotype changes Pro718 to Gly (PG). However, the PG mutation alone does not render CHIKV replicons noncytotoxic; for this a combination with either Glu117 to Lys (EK) substitution or with insertion of five amino acids after residue 647 of nsP2 (5A) is required [6,63]. These mutations, as well as Lys192 to Asn (KN) substitution which completely blocks the NTPase activity of CHIKV nsP2 [3], were introduced into CMV-P1234 (Fig 8A) and their effects on the activity of CHIKV *trans*-replicase were analyzed in U2OS cells.

Mutations associated with noncytotoxic properties of nsP2 inhibit its ability to induce shutdown of RNA polymerase II mediated transcription [28]. Thus, these mutations may increase the expression of ns proteins in cells transfected with CMV promoter-based *trans*-replicase vectors. To take this effect into account the ns protein expression was analyzed using Western blotting. From the nsP2 mutations only EK (P12^EK^34) had no apparent effect on the ns protein levels (Fig 8B). KN mutation (P12^KN^34) resulted in increased expression of all ns proteins (Fig 8B), most likely because a functional helicase/NTPase is essential for cytotoxicity and nsP2-mediated degradation of Rpb1 [28]. A similar effect was also caused by PG mutation alone (P12^PG^34) or in combination with EK mutation (P12^EKPG^34) (Fig 8B); these effects correlate with reduced cytotoxicity of CHIKV replicons harboring the PG mutation [63]. Similar, albeit less prominent, effect was also observed for 5A mutation (P12^5A^34); again this is coherent with reduced cytotoxicity of corresponding replicons [6].

It was observed that despite elevated expression of ns proteins Gluc expression in the cells co-transfected with CMV-P12^KN^34 + CMV-Fluc-Gluc did not exceed background levels (Fig 8C). This indicates that the NTPase, helicase and/or RNA triphosphatase activities, all fueled by the affected active site, were crucial for CHIKV replicase function. This finding is also in agreement with results obtained using SFV genomes [64] and *trans*-replication systems [19]. PG substitution had no negative effect on the activity of CHIKV *trans*-replication system (Fig 8C). Thus, defects in protease, NTPase and RNA helicase activities, that have been observed for recombinant nsP2 harboring the corresponding mutation in cell-free assays [6], were not detected in a cellular environment. Alternatively, the negative effect of PG substitution may, fully or partially, be compensated by elevated expression levels of ns proteins (Fig 8B). However, this would seem unlikely as elevated ns proteins levels do not necessarily result in more efficient RNA replication (Fig 3). The levels of Gluc expression in cells co-transfected with CMV-Fluc-Gluc and CMV-P12^EK^34 or CMV-P12^EKPG^34 were below 0.5% of that observed for CMV-Fluc-Gluc + CMV-P1234 co-transfected cells (Fig 8C). This correlates with the extremely low replication of the corresponding replicon RNAs [6]. Combined with the lack of effect on ns protein expression (Fig 8B) these findings suggested that the noncytotoxic phenotype of replicons harboring EK mutation most likely represents a consequence of a severe defect in RNA replication, which, in turn, leads to drastically diminished nsP2 levels [6]. Restoring the ns protein levels in the *trans*-replication system (Fig 8B) could not compensate for this replication defect. Importantly, CHIKV genomes harboring EK or EKPG mutations also have low infectivity and undergo reversions [6] e.g. display phenotype very similar to that of ICRES-4^HF^. In contrast, the 5A mutation reduced Gluc expression by CHIKV *trans*-replication system only ~2-fold (Fig 8C). CMV-P12^5APG^34, harboring a combination of mutations associated with the noncytotoxic phenotype of CHIKV replicon, was a ~5-fold less efficient inducer of Gluc expression than CMV-P1234 (Fig 8C). This confirmed that the PG and 5A mutations affect each other; accordingly their combination leads to a phenotype different from that caused by either mutation individually [6]. Most importantly, this data indicates that severe reduction of RNA replication (such as caused by EK or EKPG mutations) is not required to generate noncytotoxic CHIKV replicons. Taken together, the results obtained using CHIKV *trans*-replication system complemented and supported previous findings. Thus, the *trans*-replication system is a sensitive and reliable instrument in the functional analysis of replicase mutations.

## Discussion

Here we report the construction, properties and use of CHIKV *trans*-replication systems. These systems were designed to be robust, highly efficient/sensitive and easy to use. For this purpose template RNAs expressing Fluc and, *via* SG RNA synthesis, Gluc reporter proteins were constructed. Due to very low background levels, we saw a great boost in Gluc activity both by CMV- and T7 promoter-based *trans*-replicases (Fig 2) with an excellent correlation between Gluc activities and levels of SG RNAs in transfected cells (Figs 2A and 3B). Expression of the first reporter protein (generally Fluc; Figs 2C and 4B) was clearly boosted in the case of T7 promoter-based CHIKV *trans*-replicase (Fig 2C and 2D); as previously observed for SFV *trans*-replicase [19]. Due to high background levels, only moderate boost in the expression of Fluc by the CMV promoter-based *trans*-replicase was observed (Fig 2C and 2D). Nevertheless, the replicase produced from the CMV promoter-based vector caused equally, if not more, prominent amplification of template RNA (Fig 3B).

High efficiency, sensitivity and reproducibility of the *trans*-replicase are pre-requisites for its use in the functional analysis of mutations seriously impairing replicase activity. This is especially important because these are the very mutations that are unstable in the context of replicating RNA genomes. It was observed that the expression of ns proteins at maximal possible levels was not required for the highest activities of the *trans*-replication system (Fig 3): once very high levels of SG RNA (and Gluc reporter) expression were achieved, additional boosting of replicase expression resulted in rather small (if any) further increase of RNA synthesis. The most likely reason behind this phenomenon is negative feedback as increased levels of nsP2 are known to alter the ns polyprotein processing pathway [65], thus limiting the number of active replicase complexes per cell. The maximal activities of *trans*-replicases depended also on the cell type (Figs 2 and 3); possibly because of differences in cell size, different availability of essential host factors and/or due to the ability of the host cells to affect the stability of replicase complexes [66]. In order to retain the sensitivity of the assay, setups that were highly efficient but not over-saturated were used: most commonly U2OS cells, CMV-P1234 (or its derivatives) as standard plasmid and CMV-Fluc-Gluc as the source of template RNA.

The high sensitivity of the system enabled us to distinguish lethal mutations from those having a strong negative impact on CHIKV replicase activity. Thus, only replicases harboring mutations in the active sites of nsP4 polymerase (P1234^GAA^) or nsP2 NTPase/triphosphatase/helicase (P12^KN^34) were unable to replicate/transcribe the template RNA (Figs 3B and 8C). A number of mutations caused severe reduction, but did not abolish replicase activity (Figs 4B, 4C and 8C). It was directly shown either in this study (P1234^HF^; Fig 5D) or known from previous studies (P12^EK^34, P12 ^EKPG^34 [6]) that viruses harboring the corresponding mutations were genetically too unstable to allow any functional studies. This could also be extrapolated to mutations present in P1^E^234-A, P1^E^234-B, P1^E^234-D, P1234^E^ and P1234^HS^ (Fig 4B and 4C): their impact on CHIKV replicase activity was too prominent to allow the corresponding viruses to be genetically stable. There is likely to be a threshold level of replicase activity, necessary for the genetic stability of mutant RNA genomes. Based on the mild instability of Repl-1^E^ (Fig 5B) and moderately compromised replicase activity of T7-P1^E^234-C (Fig 4) this mutant is close to such threshold. Indeed, we have observed that virus genomes harboring the same mutation could efficiently be rescued and propagated but were not stable enough to allow multiple *in vitro* passages.

The CHIKV *trans*-replication systems can be used for multiple applications. It was shown that the phenotypes, caused by the mutations studied in the context of CHIKV replicons and genomes [6,63], were reproduced in the *trans*-replication system (Fig 8). As any defect in virus replicase proteins can cause adaptations and/or reversions, a genetically stable *trans*-replication system is clearly superior for estimating the direct consequences of such defects on the functionality of the replicase. The tagging of ns proteins in the *trans*-replication system also resulted in several intriguing questions regarding the functions of the C-terminal regions of nsP1 and nsP4. The EGFP-tag was reasonably well tolerated in one specific position of nsP1 but not in nearby positions; nsP4 apparently did not tolerate the HA-tag at its C-terminus (Fig 4). These aspects represent topics for independent studies that can provide new and important information regarding the assembly and function of CHIKV replicase.

The use of COP-5 cells having an intact type-I interferon system was crucial for the discovery of a new function of the SFV replicase in eliciting an antiviral response [47]. The CMV promoter-based version of CHIKV *trans*-replication system works well in these cells (Fig 2). Thus, activities of the replicases from two different alphaviruses can now be directly compared and the molecular basis of the ability of SFV replicase to synthesize interferon-inducing dsRNAs using cellular templates can be revealed. Furthermore, although the wild type CHIKV *trans*-replicase performed rather similarly in all the tested cell lines (Fig 2), this is not necessarily the case for its mutant forms. This provides an opportunity for detailed analysis of host-specific effects of mutations in the ns proteins (and/or template RNAs); these studies can be extended to the identification and characterization of host factors and mechanisms important for alphavirus replication. Finally, a *trans*-replication system can also be developed specifically to be used in insect cells. Interestingly, constructs expressing short template RNAs for CHIKV replicase have been previously tested both in cultivated mosquito cells and *in vivo* [67]. However, the amplification/transcription of the template RNA achieved using the previous system was less than 20-fold, much less prominent compared to at least 10^3^-fold amplification observed in the current study (Fig 2). While it is possible that the low efficiency is a property of insect cells, it is more likely that there is a lot of room for improvement in the insect cell-based system. If the levels of RNA amplification/transcription can approach those seen in mammalian cells, it would open a possibility for the development of efficient conditional expression systems for insect cells and highly sensitive reporter cell lines.

The CHIKV *trans*-replicase represents a useful system for tagging of functional ns proteins (Fig 4). Such *trans*-replicases, replicons and virus genomes represent valuable instruments for studies of virus replication and virus-host interactions. Thus, ICRES-4^SF^ (then named CHIKV LR2006-OPY1-nsP4/FLAG) has already been used to visualize nsP4, which was not detectable by nsP4-specific antibodies in CHIKV infected cells [8]. ICRES-4^SF^ can also be used for analysis of interactions between CHIKV nsP4 and cellular proteins as has been done for SINV [38]. CHIKV is clearly better suited for this analysis as individual nsP2 of CHIKV efficiently processes the cleavage site in P34 polyprotein [6]. In contrast, at late stages of SINV infection P34 remains mostly uncleaved [68] making it difficult to distinguish whether identified host proteins interact with individual nsP4 or with the nsP3 part of P34 polyprotein. Similarly, as RNA genomes harboring EGFP tag in nsP1 are stable enough to allow experiments in cell culture, host factors binding to nsP1 can be revealed by the same approach previously utilized to identify host proteins interacting with nsP2 [37] and nsP3 [36,58].

Finally, CHIKV *trans*-replication system can be applied for the development of antiviral strategies. It has already been, to some extent, used for analyzing the molecular bases of attenuation of CHIKV Δ5nsP3 virus [10], a highly efficient CHIKV vaccine candidate [69]. The *trans*-replication system is also suitable for the discovery and subsequent analysis of inhibitors affecting CHIKV replicase formation and function. Thus, with the T7 promoter-based version of the system, it can be analyzed which exact step of RNA replication (negative strand, new genomic RNA and/or SG RNA synthesis) is affected by the compounds. The *trans*-replicase based assay would also simplify identification of the precise functions affected by different inhibitors. For example, in the context of virus infection, the amounts of ns proteins (down-regulated due to the overall suppression of viral RNA synthesis) may simply be too low to enable detection of the effect of an inhibitor on e.g. ns polyprotein processing, or on the intracellular localization of viral ns proteins. Uncoupling the replicase expression from RNA replication makes such assays feasible; the availability of mutant and tagged forms of *trans*-replicase provides further advantages for such studies.
